# Endogenous and exogenous stem cells: a role in lung repair and use in airway tissue engineering and transplantation

**DOI:** 10.1186/1423-0127-17-92

**Published:** 2010-12-07

**Authors:** Dimitry A Chistiakov

**Affiliations:** 1 Department of Molecular Diagnostics, National Research Center GosNIIgenetika, 1st Dorozhny Proezd 1, 117545 Moscow, Russia

## Abstract

Rapid repair of the denuded alveolar surface after injury is a key to survival. The respiratory tract contains several sources of endogenous adult stem cells residing within the basal layer of the upper airways, within or near pulmonary neuroendocrine cell rests, at the bronchoalveolar junction, and within the alveolar epithelial surface, which contribute to the repair of the airway wall. Bone marrow-derived adult mesenchymal stem cells circulating in blood are also involved in tracheal regeneration. However, an organism is frequently incapable of repairing serious damage and defects of the respiratory tract resulting from acute trauma, lung cancers, and chronic pulmonary and airway diseases. Therefore, replacement of the tracheal tissue should be urgently considered. The shortage of donor trachea remains a major obstacle in tracheal transplantation. However, implementation of tissue engineering and stem cell therapy-based approaches helps to successfully solve this problem. To date, huge progress has been achieved in tracheal bioengineering. Several sources of stem cells have been used for transplantation and airway reconstitution in animal models with experimentally induced tracheal defects. Most tracheal tissue engineering approaches use biodegradable three-dimensional scaffolds, which are important for neotracheal formation by promoting cell attachment, cell redifferentiation, and production of the extracellular matrix. The advances in tracheal bioengineering recently resulted in successful transplantation of the world's first bioengineered trachea. Current trends in tracheal transplantation include the use of autologous cells, development of bioactive cell-free scaffolds capable of supporting activation and differentiation of host stem cells on the site of injury, with a future perspective of using human native sites as micro-niche for potentiation of the human body's site-specific response by sequential adding, boosting, permissive, and recruitment impulses.

## Introduction

Transplantation of the airway and lung tissue is an accepted modality of treatment for end-stage lung disease. Since the early 1990 s, more than 26,000 lung transplants have been performed at centers worldwide [1]. The most common indications, for which lung transplantation is performed, include cases of respiratory failure such as chronic obstructive pulmonary disease, cystic fibrosis (mucoviscidosis), idiopathic pulmonary fibrosis, idiopathic pulmonary hypertension, alpha-1 antitrypsin deficiency, bronchiestasis, and sarcoidosis [2]. However, the availability of donor tissues and organs is constantly limited, which presents a serious bottleneck for widespread transplantation surgery. The generation of bioengineered lung and tracheal tissue transplants, with the help of regenerative medicine, is considered a very promising alternative to the classical transplantation of donor organ/tissue.

Over two years ago, a successful transplantation of the world's first bioengineered trachea to a young woman with end-stage bronchomalacia was performed [3]. A donor trachea was first carefully decellularized using a soft detergent that prevented degradation and solubilization of the collagenous matrix. Major histocompatibility antigens were also removed from the donor trachea to prevent a transplant rejection reaction. The decellularized trachea was then seeded with two types of pre-expanded and predifferentiated autologous cells; i.e. mesenchymal stem cell-derived cartilage-like cells and epithelial respiratory cells. Finally, the bioengineered organ was engrafted into the recipient's body to replace the left main bronchus. After surgery, the patient did not develop any signs of antigenicity and continues to live a near-normal life.

The first tissue-engineered organ transplantation was still based on a donor trachea. However, to date, a variety of bioengineered tubular tracheal matrices were developed as an alternative to the donor's airway. When selecting new biomaterials for trachea bioengineering, researchers should evaluate a wide range of biological properties of candidate material including toxicity, toxigenicity, biocompatibility, biodegradability, durability, cell adhesion characteristics, and ability to mimic the function of a native organ as much as possible.

The epithelial cells-extracellular matrix (ECM) interactions play a crucial role in healing airway injuries and repair of the airway epithelium. The secretion of a provisional ECM, the cell-ECM relationships through epithelial receptors, and the remodeling of the ECM by matrix metalloproteinases contribute not only to airway epithelial repair by modulating epithelial cell migration and proliferation, but also to the differentiation of repairing cells, leading to the complete restoration of the wounded epithelium [4]. Therefore, while developing a bioengineered model of the human bronchiole, tissue engineers should pay special attention to the fabrication of biologically active scaffolds and matrices capable of fulfilling natural properties of the airway ECM, for example, by maintaining and slowly releasing factors essential for proliferation and differentiation of a stem cell transplant [5].

Another issue of challenge in lung regenerative medicine is the choice of an appropriate cell source to reconstitute the lung airway. Naturally, residual pools of adult stem cells (SCs) located within the basal layer of the upper airways, within or near pulmonary neuroendocrine cell rests, at the bronchoalveolar junction, and within the alveolar epithelial surface, are responsible for lung regeneration and repair [6]. Endogenous progenitor cells are also involved in lung regeneration, contributing particularly to the rapid repair of the denuded alveolar surface after injury [7,8]. However, the repair capacity of lungs declines with age, which is primarily due to the endogenous SC failure. Therefore, the exogenous stem/progenitor cells, such as embryonic stem cells (ESCs), bone marrow-or fat-derived mesenchymal stem cells (MSCs), and recently amniotic fluid stem/progenitor cells, could be considered as an alternate cell source for lung regeneration. The limitation of xenogenic or allogenic SCs is their potential immunogenicity for the recipient organism that requires implementation of immunosuppression to minimize the risk of graft rejection.

Special attention should also be paid to the delivery of implant cells to the recipient site and providing boosting and recruitment impulses for survival, expansion, and differentiation of the stem cell transplant. The molecular physiology of complex interactions between the host and engrafted cells is far to be precisely understood and therefore needs further efforts to maximize the regeneration rate. In this review, we characterize the important role of cell-cell interactions and ECM in airway epithelium repair; consider the resources of endogenous and exogenous stem/progenitor cells that have been used or have a potential to be applied in lung regeneration; and analyze current strategies in tracheal bioengineering and transplantation.

### Endogenous and exogenous stem and progenitor cells for lung repair

The airway epithelium is subjected to a lifetime exposure by inhaled particles and pathogens that may lead to the development of a variety of infectious and inflammatory respiratory diseases such as chronic bronchitis, asthma, chronic obstructive pulmonary disease, and cystic fibrosis. These pathologies are typically associated with changes in the architecture of the airway walls, which could vary from the epithelial structure remodeling to complete denudation of the basement membrane. To restore its functions, the airway epithelium has to rapidly repair the injuries and regenerate its structure and integrity. The regeneration process is a complex phenomenon that quickly starts after the lesion occurs. Epithelial cells at the wound edge dedifferentiate, spread, and migrate to cover the denuded area [9]. After migration, epithelial cells in the repairing area start to proliferate. Finally, to restore a functional mucociliary epithelium at the injury site, the epithelium forms a transitory squamous metaplasia followed by progressive redifferentiation [10].

Due to the very large size (> 70 m^2^) and spatial restrictions of the alveolar surface in adult humans, a large number of cells must function as a "ready reserve" to repair the damaged alveolar surface [11]. The repair of injuries and the regeneration of the epithelial structure involve stem and progenitor cells. The mechanism of the regeneration of the airway epithelium was widely studied in rodent models of lung injury. After epithelial damage in mouse, the sites of actively proliferating cells observed near the glandular ducts were referred to as basal cells providing evidence of SC niches [12]. These cell populations are heterogeneous and comprise subpopulations capable of either multipotent or unipotent differentiation leading to the restoration of a completely differentiated airway epithelium [13].

In the human fetus, both basal and suprabasal cells were able to reconstitute a fully differentiated airway epithelium after engraftment in a humanized xenograft model in severe combined immunodeficiency (SCID) mice suggesting for a similar progenitor potential [14]. In adult human airway epithelium, only isolated basal cells are capable of restoring a fully functional airway epithelium, but the adult secretory cells lose their regeneration potential compared to the fetal secretory cells [15]. Thus, although both secretory and basal cells are able to proliferate, only basal cells are now suggested to represent the SC compartment of the airway epithelium in tracheas and bronchi.

In rodent bronchioles, two types of cells, Clara cells and neuroendocrine cells localized in neuroepithelial bodies possess the ability to proliferate in response to bronchiolar and alveolar damages [16]. Among those, only a subset of Clara secretory protein-expressing cells, which are reside in the airway neuroepithelial bodies and bronchoalveolar duct junctions are able to reconstitute the bronchiolar airway epithelium and hence can be considered as bronchiolar SCs [17]. A population of endothelial cells resistant to bronchiolar and alveolar damages and which is capable of giving rise, not only to Clara cells, but also to type I and type II alveolar cells in vitro was found at the bronchioalveolar duct junction [18]. Indeed, this observation suggests that both bronchiolar and alveolar rodent epithelia could possess SC features.

Despite the well-documented nature of stem/progenitor cells in rodent bronchiolar airway epithelium, it remains to be clarified in humans. Recently, the multi-way analyzes of multicellular spheroids (termed as bronchospheres) produced by mechanical and enzymatic digestion of the adult human lung tissue revealed the presence of mixed phenotype cells with type II alveolar and Clara cell features and high expression of SC regulatory genes, which was either weakly or not detectable in original tissues [19]. These findings provide the evidence that adult human bronchioli, similarly to rodent bronchiolar and alveolar epithelia, should harbor SCs. Interestingly, the bronchospheres also exhibited mesenchymal features and, after silencing the Slug gene that plays a key role in epithelial-mesenchymal transition processes, they lost the SC-specific gene expression profile and gained a differentiated bronchial/alveolar phenotype [20]. This suggests that the epithelial-mesenchymal transition process could be induced in a subset of airway cells after injury of the adult human lung tissue.

The endogenous peripheral airway smooth muscle progenitors appear to occur very early in lung development. The peripheral mesenchyme that expresses fibroblast growth factor 10 (Fgf10) serves as a progenitor cell population for peripheral airway smooth muscle [21]. As the airway grows outwards, Fgf10-expressing airway smooth muscle progenitor cells spread along the expanding peripheral airway. The mesenchymal vascular progenitors (hemangioblasts) occur at the very early stages of the lung embryogenesis. Under the stimulation of vascular engothelium growth factor, which is secreted mainly by the primitive epithelium, these hemangioblasts differentiate into a capillary network surrounding the bronchial, lobar, and segmental branches of the airway [22].

Since adult human airway epithelial SCs were only recently discovered and their cultivation is still challenging, the researchers consider other sources of exogenous pluripotent SCs for airway tissue engineering, such as ESCs and MSCs. ESCs possess a great pluripotency since they are able to generate a variety of cell lineages including airway progenitor cells [23]. However, the application of human embryonic SCs is now limited due to the obvious ethical problems.

Bone marrow-derived adult MSCs circulating in blood were shown to be able to support lung repair in mice [24,25]. There were two populations of those progenitor epithelial cells expressing distinct surface markers. The first population had epithelial characteristics as shown by cytokeratin expression, but also hematopoietic characteristics as shown by CD45 expression [24]. Another population of MSCs was positive for the early epithelial marker cytokeratin 5 (CK5) and the chemokine receptor CXCR4 [25]. Administration of FGF7 (also known as a keratinocyte growth factor) to mouse recipients of tracheal transplants resulted in enhanced engraftment of the CK5-positive progenitors to the injured proximal airway epithelium suggesting the role of FGF7 in local resident progenitor epithelial cell repair through the mobilization of subsets of CK5-positive epithelial progenitors [26].

Bone marrow-derived populations of MSCs, such as bone marrow-derived chondrocytes, were widely co-cultivated with respiratory epithelial cells to reconstitute artificially fabricated trachea constructs based on synthetic [27], composite [28], or natural decellularized matrixes [3,29]. A tissue-engineered trachea seeded with bone marrow-derived chondrocytes and airway epithelial cells was successfully implanted into a human recipient [3]. At present, autologous bone marrow-derived MSCs seem to present the most popular SC type used in laryngotracheal tissue engineering. Adipose-derived MSCs may be also regarded as potentially suitable for the tracheal repair, but so far there are only a few reports about their use in the improvement of airway defects. Suzuki et al. [30] used fat-derived MSCs as part of a bioengineered scaffold to improve tracheal defects in rats, observing a well-differentiated and neovasularized airway epithelium two weeks post-implantation. A tissue-engineered trachea derived from a framed collagen scaffold, gingival fibroblasts, and adipose-derived SCs, showed good regeneration properties when implanted into rats with tracheal defects [31].

Human amniotic fluid SCs (hAFSCs) and umbilical blood cord (UBC)-derived SCs are new cell resources for lung regeneration. Human umbilical cord blood is a promising source for human MSCs. Hematopoietic SCs are present in the blood of the umbilical cord during and shortly after delivery. These SCs are in the blood at the time of delivery because they move from the liver (where blood formation takes place during fetal life) to the bone marrow (where blood is made after birth). UBC-derived SCs are similar to SCs that reside in the bone marrow. Higher healing properties of those cells were demonstrated in rodents. When administered intratracheally, human UCB-derived MSCs successfully attenuated the hyperoxia-induced lung injury in neonatal rats [32] and reduced fibrosis in the bleomycin-induced mouse model of lung injury through the activation of production of matrix metalloproteinases (MMPs) and inhibition of the impaired collagen synthesis [33]. Recently, a successful clinical application of human UCB-derived SCs for treatment of systemic lupus erythematosus-induced diffuse alveolar hemorrhage was reported. The cells were infused into the blood of a 19-year-old girl that showed dramatic improvements in her clinical condition, oxygenation level, radiographic and hematological status very soon after transplantation of MSCs [34].

Similarly to ESCs, hAFSCs are multipotent and capable of differentiating into cell types that represent each embryonic germ layer, including cells of adipogenic, osteogenic, myogenic, endothelial, neuronal, and hepatic lineages [35]. However, compared to ESCs, hAFSCs have a great advantage because they are not tumorigenic and teratogenic. Experiments in mice with lung injuries showed an excellent regeneration potential for hAFSCs, which were able to integrate into the murine lung and differentiate into pulmonary lineages after injury [36].

In 2006, an attractive possibility of the direct reprogramming of somatic cells to an embryonic stem cell-like pluripotent state was shown [37]. The reprogramming requires the ectopic expression of four or even fewer factors (Oct 4, Sox2, Nanog, and Klf4) responsible for maintaining pluripotency [38]. To maintain a pluripotency, human induced pluripotent stem (iPS) cells were shown to utilize signaling mechanisms that are similar to those used by human ESCs [39]. The iPS cells present a key advantage over true ESCs since they do not require an embryo to be sacrificed and ultimately will allow the autologous transplantation of induced SCs to repair damaged tissues [40]. To date, a variety of human and murine terminally differentiated somatic cells were reported to be reprogrammed into iPS cells. While no direct applications to airway cells have yet been reported, it is likely that such applications will become possible in the near future.

Early-passage iPSCs retained a transient epigenetic memory of their somatic cells of origin, which manifests as differential gene expression and altered differentiation capacity [41,42]. These observations could be exploited in potential therapeutic applications to enhance differentiation into desired cell lineages.

It should be noted that in vitro reprogramming of somatic cells to iPS cells occurs with extremely low frequency and slow kinetics, suggesting the existence of a barrier factor. Cell senescence modulated by the activation of several negative cell cycle regulators, such as p53 (encoded by Trp53), p21 (encoded by Cdkn1a), and INK4a/ARF (encoded by Cdkn2a/2b), was considered to play a major barrier role for reprogramming [43-45]. Before the reprogramming stage, a preliminary down-regulation of these factors resulted in a marked increase (up to 28%) in the efficiency of reprogramming [46-48]. For example, silencing of p53 significantly increased the reprogramming efficiency of human somatic cells, directed with administration of only two pluripotency factors, Oct4 and Sox2 [47]. These results suggest new routes to more efficient reprogramming, avoiding the use of oncogenes for inducing pruripotency, and maximizing yield of new promising cell sources for tissue engineering.

### Epithelial cell-extracellular matrix interactions and their mimic by bioengineered scaffolds

**Figure 1:**
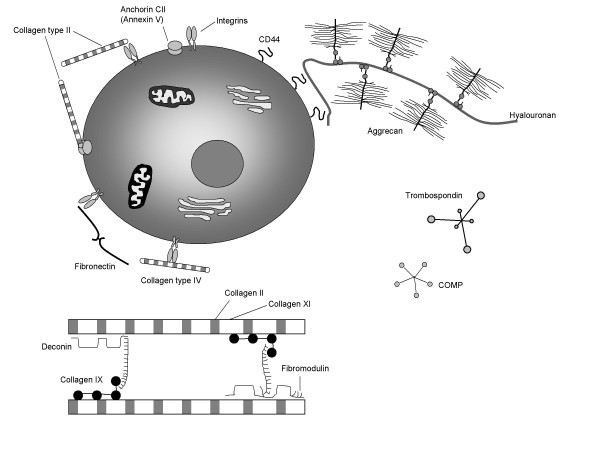
**Extracellular matrix proteins and their interaction with each other and with cell surface matrix receptors**. Decorin, fibromodulin, and types IX and XI collagen all interact with type II collagen and regulate collagen fiber assembly and structure. Types II and VI collagen bind matrix receptors (integrins). Type II collagen can also bind to anchorin CII, whereas fibronectin binds to integrins. A large proteoglycan aggregate forms when multiple aggrecan molecules bind to a long strand of hyaluronic acid, which, in turn, is anchored to the cell by CD44. Additional matrix proteins shown include thrombospondin and cartilage oligomeric protein (COMP).

The airway epithelium plays a key role in wound healing through the release of ECM proteins and remodeling of the secreted provisional ECM. The epithelial cells secrete a range of factors contributing to airway repair and regeneration (Figure 1). They include structural matrix proteins (collagens, laminin, fibronectin, fibrin, etc.) and molecules modulating cell migration (integrins), cell-cell and cell-substrate interactions (glycans, cell adhesion receptors), ECM remodeling (MMPs), cell proliferation and differentiation (FGF7, epidermal growth factor (EGF), connective tissue growth factor, FGFs, and their receptors) [4]. The epithelial cells and inflammatory cells of the airways also release inflammatory mediators such as transforming growth factor (TGF)-β_1 _and tumor necrosis factor-α that influence the production of matrix molecules [49].

Tissue engineering presents a promising technique to create a functional tracheal substitute that may overcome many difficulties that other tracheal substitutes could not. Most cartilage tissue engineering approaches use biodegradable three-dimensional (3D) scaffolds, which are important for neocartilage formation by promoting chondrocyte attachment, cell redifferentiation, and production of extracellular cartilage matrix [50]. In the development of bioactive engineered matrices, current strategies try to utilize natural properties of the airway ECM as much as possible. Those include the creation of hybrid hydrogel 3 D networks containing a cell-binding site for ligation of cell-surface integrin receptors and substrates for MMPs, proteases implicated in wound healing and tissue regeneration [51,52] and fabrication of scaffolds with immobilized signaling molecules (FGF [53], TGF-β_1_[54], etc.) that slowly release upon transplantation to support the tissue repair.

In order to increase non-integrin-dependent cell adhesion features of the engineered scaffold, using fibrin/hyaluronic acid (HA) composite as a scaffold biomaterial was suggested. HA (hyaluronan) is natively found in the cartilage tissue. This glucosaminoglycan functions as a core ECM molecule for the binding of keratin sulfate and chondroitin sulfate in forming aggrecan [55] and contributes to several cellular processes like cell proliferation, morphogenesis, inflammation, and wound repair [56]. Compared to relatively inert poly(ethylene glycol) (PEG) hydrogels, fibrin/HA gels exhibited better properties for supporting the differentiation of MSCs into chondrocytes as shown by enhanced expression of cartilage-specific markers by MSCs seeded into the fibrin/HA scaffold [57]. In humans, HA-based scaffold Hyalograft C (Fidia Advanced Biopolymers, Abano Terme, Italy) has been successfully applied in the treatment of chronic lesions of the knee and articular defect repair [58]. To date, Hyalograft C has not been used for tracheal repair in humans. The application of this scaffold for tracheal regeneration in animal models produced inconsistent results. A tissue-engineered trachea, fabricated from the fibrin/HA gel and autologous chondrocytes, and then transplanted into rabbits, showed successful regeneration and functional restoration of ciliated epithelium at the operated site without graft rejection and inflammation [59]. However, in another study, if implanted intra-or paralaryngeally, Hyalograft C exhibited biocompatibility-related problems initiating a foreign-body reaction and cartilage degradation in rabbits [60].

To avoid problems with biocompatibility, the use of scaffold-free cartilage grafts was proposed. The grafts were recently evaluated in laboratory animals. Autologous chondrocytes were cultivated in a bioreactor to fabricate scaffold-free cartilage sheets and then used for laryngotracheal reconstruction in rabbits. The scaffold-free engineered cartilage was capable to support the formation of a well-vascularized, autologous neotrachea, with excellent mechanical properties compatible with the rabbit's native trachea [61]. The grafts showed no signs of degradation or inflammatory reaction and were covered with mucosal epithelium. However, they did show signs of mechanical failure at the implantation site [62]. Overall, scaffold-free engineered cartilage represents a promising, new approach in tracheal reconstitution; however, further efforts are required to optimize its mechanical properties and biomaterial durability.

### Strategies to deliver cell graft and support its survival and tracheal healing

Pioneering works involved the engraftment of human airway epithelial cells into immunodeficient (SCID) mice resulted in the development of a well-differentiated and functional human epithelium [63,64]. Yang et al. [65] developed an approach for cultivating and scaffold-free delivery of epithelial cell sheets directly to host tissues. The utility of human respiratory epithelial cells for clinical transplantation is limited because those cells grow slowly. After transduction with a lentivirus-based vector, the growth rate and regeneration properties of transduced human epithelial cells have been significantly improved [66]. However, due to their viral modification, the clinical implication of transduced epithelial cells is still restricted by biosafety requirements.

Primary respiratory epithelial cells co-cultivated with other primary cells, such as fibroblasts and chondrocytes, and seeded into the collagenous or related carrier, were shown to reconstitute the structure of the tracheal wall, forming a fully differentiated airway epithelium and basement membrane located below the epithelium [67,68]. To date, a range of experimental approaches and protocols for the in vitro development of lung tissue constructs composed of primary lung cells has been developed. For example, the development of a tissue-engineered model of human bronchiole consisting of primary cells and designed to study mechanisms of airway remodeling and lung inflammation in asthma has been recently reported [69].

Exogenous stem/progenitor cells may be delivered into the lung either intravenously, intratracheally, or by direct injection. The immediate efficiency of exogenous cell arrival and trapping in the lung is very high. However, the rate of extravasation of implanted cells from the capillary into the injured tissue and eventual integration into lung cell lineages was very low (< 5%) [70]. Better results could be produced when stem/progenitor cells are cultured with other cells and seeded into the natural or artificial scaffold. Co-cultivation of epithelial progenitors with autologous costal chondrocytes, smooth muscle cells, and respiratory ciliated epithelium followed by propagation in the natural decellularized matrix (i.e. pig jejunal segment with its own vascular pedicle) resulted in the development of a functional vascularized trachea containing the extracellular cartilaginous matrix [71]. The experience with the engineered pig trachea was then used in engineering human trachea produced after dissemination of cartilage-like MSCs and epithelial respiratory cells in the decellularized donor trachea, and successful transplantation of this construct into the recipient woman [3].

### New trends in tissue-engineered tracheal transplantation

Huge efforts in the development of cell therapy-based approaches, tissue engineering techniques and their careful evaluation in animal models of lung diseases and induced airway injury, have yielded the first successful clinical applications in lung repair. The need for the introduction of new, efficient, and promising technologies for regeneration of tracheal and other defects in the lung tissues will increase along with the constantly rising number of people suffering from respiratory troubles.

Recent advances in airway tissue engineering provide a good opportunity for the treatment of a wide range of lung defects. In addition to the respiratory failure cases mentioned above, SC-based therapies show great potential for new clinical applications against acute respiratory distress syndrome [72], asthma [73], and bronchopulmonary dysplasia [74]. At present, the therapeutic potential of SCs is intensively assessed in rodent models of these diseases, with the possibility of proceeding to clinical trials [75-78].

The current regenerative medicine is leading to a new paradigm in medicine biotransferring heterologous concepts to the onset of autologous technologies that could lead to tissue regeneration in vivo. The new concept also includes studying and reproducing biological properties of ECM that regulates tissue differentiation in at least three ways: (i) the biochemical composition of the matrix constituents; (ii) the 3D-organization (architecture); and (iii) the mechanical forces mediated to the cells by the matrix. The in vivo ECM constitutes the biopolymer, which potentially plays a permissive role for tissue differentiation. The practical consequence of this research is the development of cell-free scaffolds capable of supporting activation and differentiation of host SCs on the site of injury.

The presence of the cell-free matrix and denuded tracheal segments stimulates the tracheal repair by attracting resident stem and non-stem epithelial cells to dedifferentiate, proliferate, expand over the denuned surface, and redifferentiate again [11]. By conjugating a collagen vitrigel membrane to a collagen sponge, Tada et al. [79] developed a bipotential collagen scaffold capable of promoting host epithelial cell growth and mesenchymal cell infiltration after transplantation to an animal model with tracheal defects. The new cell-free scaffold was successfully tested in rats and then in dogs. In the dog, the scaffold larynx implant was covered with soft tissue on day 18 post-surgery, followed by complete regeneration of the canine mucosa [80]. Recently, clinical trials showed good regeneration properties of this artificial scaffold for the repair of tracheal defects. The tracheoplasty of four patients (three with thyroid cancer and one with subglottic stenosis) with resection of the trachea and subsequent suturation of the defects with a cell-free scaffold (Marlex mesh tube covered by collagen sponge) resulted in a well-epithelialized airway lumen without any obstruction two months post-surgery [81,82].

The next step of the practical development of this new concept is the use of the human native site as a micro-niche in order to potentiate the human body's site-specific response by adding boosting, permissive, and recruitment impulses [83]. This technique is expected to be cost-and labor-effective since it assumes the avoidance of any in vitro cell replication, expansion, and differentiation. The new approach is a multi-step process that will include the development of strategies involving a sequential implementation (treatment) of factors required for rapid activation of endogenous SCs at the affected site, attraction of exogenous SCs from the host circulation system, control of the local release of inflammatory cytokines and hypoxia, cell differentiation toward the terminal stage, and ECM remodeling. This strategy requires a deep knowledge of molecular mechanisms, temporal-spatial signaling networks, and cell-cell interactions contributing to the tracheal repair, as well as a careful and strict real-time control of the regeneration process.

To stimulate the proliferation potential of local somatic and progenitor cells, the ectopic expression of pluripotency factors may be used. For example, Sox17 required for early endoderm formation is able to reinduce multipotent progenitor cell behavior in mature lung cells [84]. To promote further differentiation of stem/progenitor cells, it is necessary to down-regulate production of Sox2 (important for branching of airways) [85] and consider the application of tissue-specific growth factors such as EGF, FGF7, basic FGF [26,86]. Erythropoietin could be used as a boosting factor due to its emerging role in the repair of both hematopoietic and non-hematopoietic tissues [87,88].

A successful realization of the new concept would ultimately benefit achieving the end point of the developments in regenerative medicine, which considers organ regeneration rather than tissue repair.

## List of abbreviations

ARF: alternate reading frame, an alternative reading frame product of CDKN2A locus; a tumor suppressor gene; CD45: cluster of differentiation 45; CD45 antigen; protein tyrosine receptor-type phosphatase C; CDKN: cyclin-dependent kinase inhibitor; CK5: cytokeratin 5; CXCR4: CXC chemokine receptor 4; 3D: three-dimensional; ECM: extracellular matrix; EGF: epidermial growth factor; ESC: embryonic stem cell; FGF: fibroblast growth factor; HA: hyaluronic acid; hyaluronate; hAFSC: human amniotic fluid stem cell; INK4a: cyclin-dependent kinase inhibitor 2A (melanoma, p16, inhibits CDK4); iPS: inducible pluripotent stem cell; Klf4: Krueppel-like factor 4, a key transcription factor in maintaining pluripotency; MMP: matrix metalloproteinase; MSC: mesenchymal stem cell; Nanog: a key transcription factor in maintaining pluripotency; Oct4: Octamer-4, a homeodomain transcription factor maintaining pluripotency; SC: stem cell; SCID: severe combined immunodeficiency; Sox2: SRY (sex determining region Y)-box 2, a transcription factor; TGF: transforming growth factor; Trp53: transformation-related protein 53; a tumor suppressor gene; UBC: umbilical blood cord;

## Competing interests

The authors declare that they have no competing interests.
